# Enhancing patient comfort in varicose vein treatment through combined lidocaine and ropivacaine tumescent anesthesia

**DOI:** 10.3389/fsurg.2024.1359474

**Published:** 2024-05-09

**Authors:** Yubo Li, Tan Li

**Affiliations:** ^1^ Department of Plastic Surgery, Plastic Surgery Hospital, Chinese Academy of Medical Sciences and Peking Union Medical College, Beijing, China; ^2^ Department of Vascular Surgery, Beijing Chaoyang Hospital, Capital Medical University, Beijing, China

**Keywords:** tumescent anesthesia, radiofrequency ablation, lidocaine, ropivacaine, analgesic effects

## Abstract

**Objective:**

To compare the analgesic effects of specific tumescent anesthetic solutions composed of lidocaine, ropivacaine, or a combination of lidocaine and ropivacaine during endovenous radiofrequency ablation for the treatment of great saphenous vein varicosities.

**Method:**

This study included 149 patients with lower limb varicose veins who were admitted to our department between 2019 and 2023. The patients were randomly assigned to three groups: the lidocaine group (Group I), the ropivacaine group (Group II), and the lidocaine + ropivacaine group (Group III). Intraoperative vital signs, intraoperative and postoperative visual analog scale (VAS) pain scores, and long-term treatment outcomes were assessed using the venous clinical severity score (VCSS) based on clinical performance.

**Results:**

There were no significant differences in age, body mass index, operative time, or blood loss among the three groups (*P* ≥ 0.05). The differences in the mean arterial pressure and heart rate during surgery in Group II were significantly greater than those in Groups I and III (*P* < 0.05). The intraoperative VAS scores in Group II were higher than those in Groups I and III (*P* < 0.05) and at 8 and 12 h postoperatively. There were no significant differences in VCSS among the groups (*P* ≥ 0.05).

**Conclusion:**

The use of a tumescent anesthetic solution composed of lidocaine and ropivacaine significantly improved patient comfort during the perioperative period without affecting surgical outcomes. This formulation can be considered safe and reliable for preparing tumescent anesthesia solutions.

## Introduction

Lower-limb varicose veins are a highly prevalent clinical condition, affecting over 100 million people in China to varying degrees. With technological advancements, minimally invasive endovenous treatments have replaced high ligation and stripping as the mainstream therapeutic approach (1). Endovenous treatment involves injecting the tumescent anesthetic solution around the main trunk of the great saphenous vein to insulate it from surrounding tissues, prevent thermal injury, and enhance contact between the vein and the endovenous heat source. Additionally, tumescent anesthesia provides analgesia and hemostasis, making the injection of a tumescent solution a crucial factor in the success of endovenous treatments (2, 3).

The composition of tumescent anesthetic solutions varies widely in the current literature, with no standardized criteria. Local anesthetics are the pivotal components of tumescent anesthesia solutions (4). Lidocaine is a commonly used local anesthetic known for its rapid onset of action. However, its short duration often leads to postoperative pain during the early recovery period (5), necessitating additional oral analgesics (6). Ropivacaine, an amide-based local anesthetic, emerged in 1996, marking a significant advancement in pain management (7). Ropivacaine provides a longer duration of action than lidocaine, making it a suitable complement, but it has a comparatively slow onset (8). Thus, its combination with the faster-acting lidocaine for peripheral nerve blocks usually results in better anesthesia. To date, there have been no reports on the combination of lidocaine and ropivacaine in tumescent anesthesia for endovenous closure. Therefore, this study aimed to assess the effect of specific tumescent solutions composed of lidocaine (Group I), ropivacaine (Group II), and lidocaine + ropivacaine (Group III) based on postoperative pain levels and recovery in patients undergoing endovenous radiofrequency ablation, a widely used procedure in the field.

## Material and methods

### General information

The clinical data of patients with lower-limb varicose veins treated in our department between 2019 and 2023 were consecutively collected for this study. This study fully complies with the principle of the Declaration of Helsinki. The study protocol was approved by the Ethics Committee of Beijing Chaoyang Hospital and was registered as ChiCTR2100042894. The inclusion criteria were as follows: (1) age range: 18–70 years; (2) clinical presentation meeting clinical-etiology-anatomy-pathophysiology classification levels 2–4 (9), with lower limb venous Doppler ultrasounds indicating a main great saphenous vein diameter >3 mm; (3) history of an endovenous radiofrequency closure procedure; and (4) complete clinical and follow-up data. Exclusion criteria included: (1) presence of other lower limb venous diseases indicated by lower limb venous Doppler ultrasound; (2) previous history of lower limb venous surgery; (3) pregnancy; (4) creatinine clearance rate <60 ml/min, a history of hepatitis or cirrhosis, coagulation disorders, or severe underlying diseases affecting other organs; (5) ipsilateral limb ischemic arterial disease with an ankle-brachial index <0.8; and (6) patients with psychiatric disorders or communication impairments.

Based on the inclusion and exclusion criteria, 149 patients (149 affected limbs) were included in the study of 160 consecutive patients treated in our department between 2019 and 2023, with an attrition rate of 6.9%. These patients were divided into three groups based on the composition of the tumescent solution: lidocaine group (Group I), ropivacaine group (Group II), and lidocaine + ropivacaine group (Group III). Among them, there were 72 men and 77 women aged 23–69 years, with a body mass index range of 17.9 kg/m^2^–25.4 kg/m^2^. The classification based on clinical-etiology-anatomy-pathophysiology identified 49 limbs as C2, 54 as C3, and 46 as C4. The specific group allocations are shown in Table 1.

**Table 1:** Statistical analysis of individual information.

	Lidocaine (I) (*n* = 48)	Ropivacaine (II) (*n* = 52)	Lidocaine plus ropivacaine (III) (*n* = 49)	*P*-value
Sex (male/female)	23/25	25/27	24/25	*P* ≥ 0.05
Age	46 ± 23	42 ± 18	45 ± 22	*P* ≥ 0.05
BMI (kg/m^2^)	21.5 ± 3.6	22.0 ± 3.9	21.1 ± 4.3	*P* ≥ 0.05
MAP	82.9 ± 5.7	81.1 ± 4.9	83.2 ± 6.3	*P* ≥ 0.05
Preoperative heart rate	80.5 ± 12.9	76.1 ± 9.4	77.5 ± 9.9	*P* ≥ 0.05

There were no significant differences in sex ratio, age, BMI, MAP, and heart rate among all groups.

BMI, body mass index; MAP, mean arterial pressure.

### Preparation of tumescent solution

In Group I, 2,000 ml of 0.9% saline solution, 30 ml of 2% lidocaine, 40 ml of 5% sodium bicarbonate, and 2 ml of (1:200,000) epinephrine were administered. In Group II, 2,000 ml of 0.9% saline solution, 30 ml of 1% ropivacaine, 40 ml of 5% sodium bicarbonate, and 2 ml of (1:200,000) epinephrine were administered. Group III received 2,000 ml of 0.9% saline solution, 15 ml of 2% lidocaine, 15 ml of 1% ropivacaine, 40 ml of 5% sodium bicarbonate, and 2 ml of epinephrine (1:200, 000). The tumescent solution was heated to 37 °C before the procedure.

### Surgical procedure

All surgical procedures were conducted by a highly experienced vascular surgeon, consistently without the use of general anesthesia. Throughout the surgeries, an anesthesiologist remained present to monitor the patient's vital signs via a monitoring system. Preoperatively, varicose veins were marked on the patient in the standing position. After disinfection, the patient was placed in a supine position, and local anesthesia with lidocaine was administered at the puncture site above the knee joint. Under ultrasound guidance, the great saphenous vein was punctured, and a 5F sheath was introduced. The radiofrequency catheter (ERA-G5, Acotec) was subsequently introduced into the main great saphenous vein and meticulously guided to a position 1.5 cm below the saphenofemoral junction. Utilizing ultrasound guidance, the tumescent solution was then precisely injected around the main great saphenous vein and clustered branching lesions, effectively isolating the vein from surrounding tissues. It is crucial to ensure that the tumescent solution effectively and completely separates the diseased blood vessel from the surrounding tissue, rather than simply applying it superficially and waiting for 10 min. This meticulous approach helps to optimize the procedure's efficacy and minimize the risk of complications. Radiofrequency ablation was initiated, and the catheter was slowly withdrawn at a rate of 7 cm every 20 s with an operating temperature of 120 °C. Finally, Trivex (Smith-Nephew, USA) was used to treat a few clustered branch varicosities. The patients were fitted with compression stockings immediately following the procedure with a pressure range of 21–30 mmHg. To improve postoperative comfort and sleep quality on the night of surgery, patients were routinely prescribed nonsteroidal anti-inflammatory drug analgesics (Loxoprofen Sodium, 60 mg) after dinner. All patients were discharged the next day after surgery.

### Scoring

During the procedure, the mean arterial pressure (MAP), heart rate, and amount of blood loss were monitored, and the differences in MAP and heart rate between intraoperative and preoperative measurements were calculated. Visual analog scale (VAS) pain scores were recorded during the radiofrequency operation and at 2, 4, 8, and 12 h postoperatively. Anesthesiologists recorded MAP and intraoperative VAS scores, and postoperative VAS scores were recorded by ward nurses. Clinical presentations were assessed using the venous clinical severity score (VCSS) before surgery and at the 3-month follow-up.

### Statistical analysis

Continuous data were presented as mean ± standard deviation (X ± S), and between-group comparisons were analyzed using analysis of variance with the Tukey test. Categorical data were expressed as *n* (%), and the chi-square test was used for statistical analysis. The ordered data were represented as median (interquartile range) and assessed using the Kruskal–Wallis test. Dunn's multiple comparisons test was employed to analyze discrepancies among groups if the *p*-value was less than 0.05. Data were analyzed using GraphPad Prism 8.0 software, with a *p*-value of <0.05 considered statistically significant.

## Results

The three groups of patients included in the study showed no significant differences in terms of sex, age, body mass index, MAP, or heart rate (*P* < 0.05) (Table 1). Intraoperative monitoring showed that the differences in MAP and heart rate in Group II were significantly higher than those in Groups I and III (*P* < 0.05). In contrast, there were no significant differences in blood loss or operation time among the three groups (*P* ≥ 0.05) (Table 2). The VAS scores of Group II were significantly higher than those of Groups I and III during the surgery (*P* < 0.05), whereas there was no significant difference between Groups I and III. There were no significant differences in VAS scores among the three groups (*P* ≥ 0.05) at 2 and 4 h postoperatively. However, at 8 h postoperatively, the VAS score for Group I was significantly higher than those of Groups II and III (*P* < 0.05), a trend which persisted at 12 h postoperatively. There was no significant difference in the VAS scores between Groups II and III (Table 3). The VCSS showed no significant differences among groups, either before surgery or at the 3-month follow-up (*P* ≥ 0.05) (Table 4).

**Table 2:** Intraoperative monitoring of clinical parameters.

	MAP difference (mmHg)	Heart rate difference (times/min)	Blood loss (ml)	Operation time (min)	Tumescent anesthetic solution (ml)
I	8.7 ± 2.0	12.2 ± 2.6	20.1 ± 3.5	38.4 ± 9.6	581 ± 92
II	10.2 ± 3.7	14.8 ± 4.1	19.0 ± 4.7	39.6 ± 10.8	603 ± 85
III	7.9 ± 2.3	11.5 ± 2.4	18.6 ± 4.9	40.1 ± 8.2	592 ± 80
I vs. II	***P*** * * ***=*** * * **0.022**	***P**** ***<** **0.01**	*P ≥ *0.05	*P ≥ *0.05	*P ≥ *0.05
I vs. III	*P ≥ *0.05	*P ≥ *0.05	*P ≥ *0.05	*P ≥ *0.05	*P ≥ *0.05
II vs. III	***P**** ***<** **0.01**	***P**** ***<** **0.01**	*P ≥ *0.05	*P ≥ *0.05	*P ≥ *0.05

Intraoperative monitoring showed that the changes in mean arterial pressure and heart rate difference in Group II were significantly higher than those in Groups I and III. The above indices of Group II were also significantly higher than those of Group III. There was no significant difference in blood loss and the dosage of the tumescent anesthetic solution among the three groups.

Statistically significant *P*-values are in bold.

**Table 3:** Intraoperative and postoperative visual analog scale (VAS) pain scores.

	VAS scores				
	Intraoperative	2 h post-operation	4 h post-operation	8 h post-operation	12 h post-operation
I	2.5 (2.0, 3.0)	2.0 (1.5, 2.0)	2.0 (2.0, 3.0)	3.5 (3.0, 4.0)	3 (2.5, 3.5)
II	3.0 (2.5, 3.5)	1.5 (1.5, 2.0)	2.0 (2.0, 2.5)	3.0 (2.0, 3.5)	3.0 (2.0, 3.0)
III	2.0 (2.0, 3.0)	2.0 (1.5, 2.0)	2.0 (1.5, 3.0)	3.0 (2.0,3.0)	3.0 (2.0, 3.0)
I vs. II	***P**** ***<** **0.01**	*P ≥ *0.05	*P ≥ *0.05	***P**** ***=** **0.01**	***P**** ***<** **0.01**
I vs. III	*P ≥ *0.05	*P ≥ *0.05	*P ≥ *0.05	***P**** ***<** **0.01**	***P**** ***<** **0.05**
II vs. III	***P**** ***<** **0.01**	*P ≥ *0.05	*P ≥ *0.05	*P ≥ *0.05	*P ≥ *0.05

Intraoperative VAS scores in Groups I and III were significantly lower than those in Group II. The VAS scores of Groups II and III were significantly lower than those of Group I at 8 and 12 h postoperatively.

Statistically significant *P*-values are in bold.

**Table 4:** Preoperative and postoperative venous clinical severity score (VCSS).

	VCSS	
	Preoperative	Postoperative
I	16.24 ± 2.82	4.41 ± 0.52
II	16.51 ± 2.33	4.65 ± 0.55
III	16.42 ± 2.70	4.71 ± 0.61
*P*	*P ≥ *0.05	*P ≥ *0.05

There were no significant differences in VCSS among the three groups before and 3 months after surgery.

## Discussion

Postoperative pain is one of the primary concerns for patients undergoing surgery and often deters them from choosing surgical treatment for great saphenous vein varicosities (10). Therefore, studies have focused on identifying effective ways to alleviate postoperative pain. Tumescent anesthesia, first used by Klein in the 1990s, has been widely adopted in various surgical fields and has proven effective in mitigating intraoperative and postoperative pain (11). Lidocaine and ropivacaine are amide-type local anesthetics. Lidocaine, the most commonly used local anesthetic, has a rapid onset of action. However, it has a relatively short duration of action, usually lasting only 2–3 h, which may result in significant postoperative pain (12). In contrast, ropivacaine is highly bound to the protein and is more long-lasting than lidocaine (13). Moreover, it has the property of constricting blood vessels, which can delay the absorption of drugs, so it can theoretically provide patients with longer pain relief after surgery (14). Ropivacaine has been used as an alternative to lidocaine in several clinical applications, such as epidural anesthesia, nerve block, etc. (15, 16), to maintain longer analgesic effects. However, there is still a lack of research reports investigating the utilization of tumescent anesthetic solutions featuring ropivacaine as the primary component for treating varicose veins of the lower limbs.

Hence, this study hypothesized that, by combining lidocaine and ropivacaine, it would be possible to extend the duration of the tumescent solution, ultimately alleviating postoperative pain in the short term.

This study assessed pain using VAS scores for the three groups of patients. The results demonstrated that the group receiving ropivacaine alone experienced more intense intraoperative pain than the other two groups. There were no significant differences in the VAS scores among the groups at 2 and 4 h postoperatively. Starting at 8 h postoperatively, the VAS scores in Groups II and III were significantly lower than those in Group I. This trend continued up to 12 h after surgery. Roos et al. (17) revealed that for patients undergoing radiofrequency ablation of the great saphenous vein, injecting a tumescent solution with 300 ml of lidocaine, which was the local anesthetic component, resulted in an average VAS score of 2.0 at 24 h postoperatively, which aligns with the findings of this study.

Ropivacaine has also been shown to exert significantly prolonged analgesic effects. Tijanic et al. (18) reported a success rate of 96.6% for 0.75% ropivacaine regional anesthesia, which exceeded the anesthetic quality and onset time for 0.5% bupivacaine. Furthermore, ropivacaine resulted in significantly less intraoperative pain than bupivacaine. When a mixture of ropivacaine and lidocaine was used in the tumescent solution, the median duration of analgesia reached 582 min, with the longest duration extending up to 26 h, which was significantly longer than the analgesic effect of pure lidocaine (19). In this study, epinephrine was added to the tumescent solution in all of the groups. Epinephrine causes local vasoconstriction and delays the systemic absorption of local anesthetics. Research has shown that subcutaneous injection of 300 mg of ropivacaine containing epinephrine results in a maximum median peak level of 0.4 mg in the circulatory system after approximately 7 h, which is considerably later than that achieved with a tumescent solution without epinephrine. Furthermore, epinephrine does not affect the onset time of ropivacaine (20, 21). Based on the literature and the analysis conducted in this study, Group III demonstrated the advantage of a faster onset associated with lidocaine during surgery, leading to lower VAS scores compared with those of Group II, which received only ropivacaine. However, between 8 and 12 h post-operation, the prolonged action of ropivacaine, as opposed to lidocaine, enabled Group III to sustain comparatively stronger analgesic effects than Group I. This discrepancy can be attributed to ropivacaine’s prolonged anesthetic effects lasting longer than those of lidocaine. Moreover, the presence of adrenaline in the tumescent solution contributed to delaying the absorption of the anesthetic. Consequently, a portion of the tumescent solution remained within the tissues, prolonging the analgesic effect of ropivacaine post-operation. Hence, the utilization of a tumescent solution containing ropivacaine resulted in a sustained and longer-lasting analgesic effect.

Patients in all three groups exhibited stable vital signs intraoperatively, indicating that the various ratios of tumescent solutions provided satisfactory analgesic effects, and all proved to be safe and effective. Ropivacaine is commonly used for regional anesthesia, and the literature records single doses of up to 400 mg (22). Thus, the dosages in this study fall within the safe range. The intraoperative MAP and heart rate fluctuations in Group II were larger than those in the other two groups, suggesting that the onset time of ropivacaine was slower than that of lidocaine. Therefore, ropivacaine alone had a worse short-term analgesic effect during surgery than the other two agents. Levin et al. indicated that under general anesthesia and cervical head clamping, heart rate and blood pressure increase by 110% and 150%, respectively. Subsequent local infiltration of mepivacaine or lidocaine eliminated the increase in heart rate and blood pressure (23). Studies have also shown that the combined local injection of equal amounts of 2% lidocaine and 0.75% ropivacaine can significantly reduce the increase in heart rate and blood pressure during skin incision. In these studies, the blood drug concentration remained within safe levels, which is consistent with this study's findings.

Clinical symptoms of varicose great saphenous veins were evaluated using VCSS. There were no significant differences in the preoperative VCSS among the groups. Furthermore, at the 3-month follow-up, there were no significant differences in the VCSS among the groups, although all groups showed significant improvements compared to their preoperative scores, consistent with other reports (24). Additionally, there were no significant differences among the groups in terms of postoperative bleeding volume, surgical duration, or other parameters, indicating that different ratios of tumescent anesthetic solutions only affected the degree of pain during the perioperative period without affecting the surgical outcomes.

This study has some limitations. First, this was a retrospective study, and the level of evidence in evidence-based medicine was relatively low. Further randomized controlled trials are necessary to assess the reliability of these results. Second, the sample size of this study was relatively small and should be expanded for more robust conclusions. Third, this study involved a limited number of surgeons, which may have affected the generalizability of the results. In the future, involving a larger number of surgeons would be beneficial for assessing the applicability of these results.

## Conclusion

Endovenous radiofrequency ablation procedures for treating great saphenous varicose veins using a tumescent solution composed of lidocaine and ropivacaine provide reliable analgesia for patients, both intraoperatively and postoperatively, for an extended duration. This significantly improves patient comfort during the perioperative period without compromising surgical outcomes, making it a safe and reliable formulation for tumescent anesthesia.

## Data Availability

The original contributions presented in the study are included in the article/Supplementary Material, further inquiries can be directed to the corresponding author.
